# Dr. Hamilton E. Smith

**Published:** 1905-01

**Authors:** 


Dr. Hamilton E. Smith, of Detroit, Mich., died suddenly at his home October 8, 1904, of reputed heart failure. He was a graduate of the University of Buffalo, class of 1859, and was a native of this city, the date of his birth being January 22, 1840. His academic education was received at Victoria College, Toronto, Ont. During the civil war he served, first as assistant surgeon and later as surgeon of the 27th Infantry Michigan volunteers. After his muster out of service he located at Detroit, where he practised his profession until his death.

Dr. Smith attended the reunion of his class on commencement day, April 25, 1899. He was a tall, fine looking man, and at that time presented the appearance of robust health, with all the characteristics of longevity. In some pen pictures which the Journal gave of the meeting, we find the following:

Hamilton E. Smith, of Detroit, came down from Pingreeville and reunioned with the only other member of the class of '59, who was young enough to get out—Dr. W. W. Potter. This class reunion was a marvel of parliamentary procedure. There has for a year past been a very well-defined idea that the class of '98 was the wildest bunch of students ever taught to be selfrespecting doctors in the University of Buffalo. Dr. Smith told some stories of student days in the '50's, which made a couple of '98 men who had heard them feel that '98 wasn't the only red ink year on the university calendar. Dr. Smith and Dr. Potter, who both served as chief surgeons in the Union army during the '61-5 trouble, entertained a great many friends with stories of military surgery and experience.

